# Vitamin D and thyroid function: A mendelian randomization study

**DOI:** 10.1371/journal.pone.0304253

**Published:** 2024-06-20

**Authors:** Nikolina Pleić, Mirjana Babić Leko, Ivana Gunjača, Tatijana Zemunik

**Affiliations:** Department of Medical Biology, University of Split, School of Medicine, Split, Croatia; Wuhan Mental Health Centre, CHINA

## Abstract

**Background:**

Numerous organs, including the thyroid gland, depend on vitamin D to function normally. Insufficient levels of serum 25-hydroxyvitamin D [25(OH)D] are seen as a potential factor contributing to the emergence of several thyroid disorders, however, the causal relationship remains unclear. Here we use a Mendelian randomization (MR) approach to investigate the causal effect of serum 25(OH)D concentration on the indicators of thyroid function.

**Methods:**

We conducted a two-sample MR analysis utilizing summary data from the most extensive genome-wide association studies (GWAS) of serum 25(OH)D concentration (n = 443,734 and 417,580), thyroid-stimulating hormone (TSH, n = 271,040), free thyroxine (fT4, n = 119,120), free triiodothyronine (fT3, n = 59,061), total triiodothyronine (TT3, n = 15,829), as well as thyroid peroxidase antibody levels and positivity (TPOAb, n = 12,353 and n = 18,297), low TSH (n = 153,241), high TSH (n = 141,549), autoimmune hypothyroidism (n = 287,247) and autoimmune hyperthyroidism (n = 257,552). The primary analysis was conducted using the multiplicative random-effects inverse variance weighted (IVW) method. The weighted mode, weighted median, MR-Egger, MR-PRESSO, and Causal Analysis Using Summary Effect estimates (CAUSE) were used in the sensitivity analysis.

**Results:**

The IVW, as well as MR Egger and CAUSE analysis, showed a suggestive causal effect of 25(OH)D concentration on high TSH. Each 1 SD increase in serum 25(OH)D concentration was associated with a 12% decrease in the risk of high TSH (p = 0.02). Additionally, in the MR Egger and CAUSE analysis, we found a suggestive causal effect of 25(OH)D concentration on autoimmune hypothyroidism. Specifically, each 1 SD increase in serum 25(OH)D concentration was associated with a 16.34% decrease in the risk of autoimmune hypothyroidism (p = 0.02).

**Conclusions:**

Our results support a suggestive causal effect which was negative in direction across all methods used, meaning that higher genetically predicted vitamin D concentration possibly lowers the odds of having high TSH or autoimmune hypothyroidism. Other thyroid parameters were not causally influenced by vitamin D serum concentration.

## 1. Introduction

The human body receives vitamin D through synthesis in the skin after exposure to sunlight (a major source of vitamin D) or by the intake from food (only 5–10% of vitamin D is acquired in this way) [1]. Vitamin D comes in five different forms (vitamin D1-D5). The two most important of these fat-soluble secosteroids for humans are vitamin D2 (ergocalciferol) and vitamin D3 (cholecalciferol). Calcitriol (also known as 1α,25-dihydroxyvitamin D (1,25(OH)2D), 1α,25-dihydroxyvitamin D3 or 1,25-dihydroxycholecalciferol) is the active form of vitamin D3 produced in the liver from calcidiol (also known as 25-hydroxyvitamin D [25(OH)D] or calcifediol) by the action of the enzyme vitamin D 25-hydroxylase [2]. Calcitriol has several important functions, such as immunosuppressive and anticancer effects [3, 4], and it is involved in the regulation of phosphate and calcium levels, bone mineralization [5], neuromuscular and immune functions, regulation of cell growth, and the expression of more than 1000 genes [6, 7].

The influence of diet on the functioning of the thyroid gland has been extensively documented in the literature. The well-known causal influence of iodine and selenium deficiency on thyroid dysfunction has motivated numerous studies on the effect of iodine and selenium supplementation on thyroid health [8–11], however, access to vitamin D (either from food or sunlight) is also crucial for the proper functioning of the thyroid gland. Meta-analyses [12, 13] and observational studies (reviewed in Babić Leko et al. [14]) have shown that vitamin D insufficiency may be a risk factor for the development of hypothyroidism, autoimmune thyroid disorders and thyroid cancer. A large longitudinal case-control study based on electronic health records has found that vitamin D supplementation resulted in an overall decrease in TSH levels and lower rates of hypothyroidism detection [15]. A recent randomized controlled trial (RCT) has found that vitamin D supplementation reduced the incidence of hypothyroidism in females, but not in males [16]. Moreover, RCTs examining the impact of vitamin D supplementation on thyroid function in those suffering from autoimmune thyroid diseases found a decrease in anti-thyroid antibodies following vitamin D supplementation (reviewed in [14]).

The relationship between vitamin D and thyroid, however, is still poorly understood in many respects. Namely, due to the many inconsistencies between the studies (reviewed in [14]), it is still not clear how vitamin D affects thyroid hormones, thyroid stimulating hormone (TSH) and anti-thyroid antibody levels. Prior to implementing these findings in a clinical setting, it is essential to conduct additional research into the causal relationships that underpin these associations. The use of different assays to measure serum 25(OH)D levels and the possible confounding effects of age, sex, dietary practices, body mass index (BMI), smoking habits, and the season during which samples were taken are among the variables that could significantly contribute to discrepancies between the studies.

An approach that can further exclude the effect of the confounding factors, is Mendelian randomization (MR), which utilizes genetic variants as instrumental variables in order to estimate the causal effect of exposure on an outcome of interest. It is considered that this kind of methodology can mimic a randomized controlled trial [17, 18]. To date, only two studies using MR methodology investigated the association between vitamin D (mainly determined by serum calcidiol 25(OH)D levels) and thyroid function. MR methodology was used to investigate the association between serum 25(OH)D concentration and the risk of thyroid cancer, hypothyroidism, and hyperthyroidism [19], and serum 25(OH)D concentration and serum thyroid peroxidase antibodies (TPOAb) (the study included participants from China) [20]. Additionally, a recent preprint [21] has investigated the association between serum 25(OH)D concentration and the risk of hypothyroidism, Hashimoto’s thyroiditis and the levels of thyroid-stimulating hormone (TSH) and free thyroxine (fT4) using MR. However, to date, the association between serum 25(OH)D concentration and anti-thyroid antibody levels has not been investigated in populations of European ancestry. Furthermore, the associations between serum 25(OH)D concentration and the levels of free triiodothyronine (fT3) and total triiodothyronine (TT3) have not been investigated using MR methodology. Thus, the aim of this study was to assess the causal effect of serum 25(OH)D concentration on a comprehensive set of thyroid function parameters using MR methodology and the largest genome-wide association study (GWAS) summary statistics published to date. We investigated the following indicators of thyroid function; reference-range TSH, fT4, fT3, TT3, low TSH (levels below cohort-specific reference range), high TSH (levels above cohort-specific reference range), autoimmune hypothyroidism, autoimmune hyperthyroidism, TPOAb levels and TPOAb positivity.

## 2. Materials and methods

### 2.1. Study design

We conducted a two-sample MR study using GWAS summary data to examine the possible causal effects of genetically predicted vitamin D levels on thyroid function and thyroid disorders. We leveraged extensive GWAS summary data for reference-range TSH, fT4, fT3, TT3, low TSH, high TSH, TPOAb, TPOAb positivity, autoimmune hypothyroidism and autoimmune hyperthyroidism (Table 1). Our methodological approach follows the one of our previous publication [22].

**Table 1:** Characteristics of cohorts and consortia.

Trait	Consortium	Sample size (N)	Population	Reference
**Exposure**				
25(OH)D levels	UK Biobank	443,734	European	Manousaki et al. [23]
25(OH)D levels	UK Biobank	417,580	European	Revez et al. [24]
**Outcome**				
TSH	ThyroidOmics	271,040	European	Sterenborg et al. [25]
fT4	ThyroidOmics	119,120	European	Sterenborg et al. [25]
fT3	ThyroidOmics	59,061	European	Sterenborg et al. [25]
TT3	ThyroidOmics	15,829	European	Sterenborg et al. [25]
Low TSH	ThyroidOmics	153,241	European	Sterenborg et al. [25]
High TSH	ThyroidOmics	141,549	European	Sterenborg et al. [25]
TPOAb levels	ThyroidOmics	12,353	European	Medici et al. [26]
TPOAb positivity	ThyroidOmics	18,297	European	Medici et al. [26]
Autoimmune hypothyroidism	FinnGen	287,247	European	Kurki et al. [27]
Autoimmune hyperthyroidism	FinnGen	257,552	European	Kurki et al. [27]

All summary statistics are according to the HG19/GRCh37 build. All studies included both males and females of European ancestry. 25-hydroxyvitamin D, (25(OH)D); fT4, free thyroxine; fT3, free triiodothyronine; TPOAb, thyroid peroxidase antibody; TSH, thyroid-stimulating hormone; TT3, total triiodothyronine.

For every causal relationship we examined, we used data from two independent but homogenous European ancestry populations. Our study encompassed a total of 20 MR analyses, aiming to shed light on the relationship between 25(OH)D concentration and thyroid function. Our research methods and reporting align with the STROBE-MR guidelines, as detailed in S3 Appendix. Our study did not need ethical approval given that our investigation used publicly available summary statistics.

### 2.2. Data Sources for Vitamin D

Two large GWAS on serum 25(OH)D concentration were published in 2020, a month apart from each other. Both of them utilized the UK Biobank data, leading to 417,580 participants in the Revez et al. [24] analysis and 443,734 participants in Manonusaki et al. [23], as the latter one also included an additional cohort of European descent participants. In order to exclude the bias relating to the choice of the GWAS, we have performed our analyses both with Revez et al. [24] and Manousaki et al. [23] GWAS. Both studies utilized a linear mixed model for the association analysis, however, the covariates and the transformations used differed significantly between the two studies (detailed in S1 Appendix). To estimate the level of agreement between the results of the two GWAS, we extracted the mutual instrumental SNPs and calculated the correlation coefficients for the beta estimates and p-values. Given that the summary association data for 25(OH)D concentration from both GWAS were missing rsIDs, we have imputed this information using the SumStatsRehab program [28].

### 2.3. Data sources for thyroid parameters

We utilized summary association data for reference-range TSH, fT4, fT3, TT3, low TSH and high TSH from the latest and most comprehensive GWAS meta-analysis on thyroid function from the ThyroidOmics Consortium [25]. A comprehensive overview of the study cohorts and methodologies adopted can be found in Sterenborg et al. [25] and S1 Appendix. Summary association data for TPOAb levels and TPOAb positivity were obtained from an earlier ThyroidOmics consortium GWAS [26].

Autoimmune hypothyroidism and autoimmune hyperthyroidism GWAS summary statistics were obtained from the FinnGen consortium [27]. The phenotypes used in this study were “hypothyroidism, strict autoimmune” and “autoimmune hyperthyroidism”. FinnGen GWAS on hypothyroidism included 287,247 Finnish adults, of which 36,321 were cases and 250,926 were controls. FinnGen GWAS on hyperthyroidism included 257,552 Finnish adults, of which 1,621 were cases and 255,931 were controls. Participants classified as having autoimmune hypothyroidism included those who already received treatment, however, the same information was not present for participants in the autoimmune hyperthyroidism group.

### 2.4. Two-sample mendelian randomization analysis

In order to confirm the significance of the instrument’s association with the exposure, we calculated the share of trait variance explained by the genetic instruments found (R^2^) and evaluated the strength of these instruments using the F statistic.

The datasets for the exposure and outcome were aligned to ensure that the impact of identified single nucleotide polymorphisms (SNPs) on both the exposure and outcome was associated with the same effect allele. We used the Mendelian Randomization Pleiotropy RESidual Sum and Outlier (MR-PRESSO) technique for each combination of exposure and outcome to account for uncorrelated horizontal pleiotropy [29]. After removing the SNPs that were shown to be horizontal pleiotropic outliers, we recalculated the causal relationship between the exposure and the outcome.

Our primary technique of analysis was a two-sample MR analysis using the multiplicative random effects inverse variance weighted (IVW) method [30]. This method combines the influence of exposure on the outcome across various genetic variants and includes an overdispersion parameter into the variance of the (IVW) estimate, allowing the variance to expand in the presence of heterogeneity. While accommodating for heterogeneity among the SNP estimates, the multiplicative random effects IVW technique yields effect point estimates that are identical to those of the fixed effect IVW model [31].

### 2.5. Sensitivity analysis

For MR to produce valid causal inferences, the genetic instruments must satisfy three critical assumptions of instrumental variables: 1) Relevance, indicating a robust link between the instruments and the exposure; 2) Exchangeability, ensuring the instruments do not correlate with confounders that could affect both the exposure and the outcome; and 3) Exclusion, confirming that the instruments affect the outcome exclusively through their effect on the exposure [18]. To verify our adherence to the relevance assumption, we computed the R^2^ and the F-statistic for each exposure. Additionally, we used Cochran’s Q statistic for each pair of exposure and outcome to check for heterogeneity in the SNP-specific estimates.

In addition to the primary multiplicative random-effects IVW approach, we validated our findings through MR analysis employing various alternative methods, each based on distinct model assumptions: 1) the weighted median 2) the weighted mode, and 3) the MR-Egger method. The weighted median method yields a reliable estimate when at least 50% of the weight is derived from valid instruments, making it resilient to outliers among the instruments [32]. The weighted mode method operates on the assumption that the association estimate occurring most frequently is not influenced by pleiotropy, implying that it reflects the actual causal effect [33]. We produced scatter plots to depict the impact of SNPs on the exposure in comparison to their impact on the outcome for each pair of exposure and outcome, utilizing the ’TwoSampleMR’ package in R [34]. Moreover, for each pair of exposure and outcome, we carried out the MR Steiger directionality test, which assesses whether the variance accounted for by the instrumental SNPs in the outcome is smaller than that in the exposure, to confirm the reliability of the directional analysis [35]. We carried out this test using the ’directionality_test’ function within the ’TwoSampleMR’ R package.

In addition to other methods, we utilized the Causal Analysis using Summary Effect Estimates (CAUSE) method [36], a Bayesian MR approach which, in contrast to traditional MR methods, utilizes the summary association estimates of all genome-wide variants available. CAUSE effectively addresses both uncorrelated horizontal pleiotropy (where a variant independently influences both outcome and exposure) and correlated horizontal pleiotropy (where a variant affects both the exposure and the outcome through a shared heritable factor). This approach makes the assumption that a certain percentage, denoted by q, of the variants exhibit correlated pleiotropy and that all other variants have uncorrelated pleiotropic effects. As a prior on q, this assumption is included in the model. Estimates of the posterior distribution for the sharing model and the causal model are generated using CAUSE. It then establishes whether the observed relationship between exposure and outcome is more likely the consequence of causality than correlated horizontal pleiotropy by determining whether the data fits the causal model more closely than the sharing model. Differing from other MR techniques, CAUSE utilizes complete summary association data from all variants, applying linkage disequilibrium pruning at at r^2^ < 0.01 with P < 1 × 10⁻^3^, thus enhancing the analytical power of MR analysis.

In all MR analyses, a two-sided p < 0.05 was considered statistically significant. We did not adjust for the family-wise error rate (FWER) in a traditional frequentist approach, as our selection was based on a specific subset of thyroid function parameters, and we further examined the presumed causal effects through a Bayesian framework.

## 3. Results

On average, the exposure analyses with the Manousaki et al. GWAS utilized 19.6 fewer instrumental SNPs (ranging from 27 to 89) than the exposure analyses with the Revez et al. GWAS (ranging from 38 to 113). The number of common instruments present in both exposure datasets was 33. Pearson’s correlation coefficient between the beta estimates of these SNPs was equal to 0.44 (p = 0.01) while Kendall’s correlation coefficient between the p-values of these SNPs was equal to 0.72 (5.58×10^−11^). Because of the differences in methodological approaches and differences in obtained variant effects, we assumed that the subsequent MR analyses with these two exposure datasets would yield different results.

### 3.1. Serum 25(OH)D concentration from Manousaki et al. GWAS as exposure

We found a total of 109 independent SNPs for instrumental variables, which collectively accounted for 2.68% of the phenotypic variation (R^2^) in 25(OH)D concentration, as detailed in S1 Table in S2 Appendix. Each chosen SNP had F-statistics above 10, with a median of 42.25 and a range of 34.60–64.27. Following the harmonization process, two SNPs were removed from all analyses due to being palindromic with intermediate allele frequencies, while an additional SNP was removed from the TPOAb analyses as noted in S2 Table in S2 Appendix. Additionally, SNPs suspected of potential pleiotropy were excluded based on the MR-PRESSO results, as indicated in S5 Table in S2 Appendix. Consequently, the remaining instrumental variants for the MR analysis of serum 25(OH)D concentration with TSH, fT4, fT3, TT3, low TSH, high TSH, autoimmune hypothyroidism, autoimmune hyperthyroidism, TPOAb levels and TPOAb positivity were 83, 83, 86, 86, 93, 89, 87, 89, 37 and 27 respectively, as listed in S6 Table in S2 Appendix.

The multiplicative random-effects IVW MR analysis indicated a causal link between genetically predicted serum 25(OH)D concentration and hyperthyroidism (S6 Table in S2 Appendix). Nonetheless, this causal relationship was not confirmed by sensitivity analyses or CAUSE analysis. The latter, by incorporating all genome-wide SNPs, showed that the causal model did not fit the data better than the sharing model (delta_elpd = 0.54 > 0, p = 0.78).

### 3.2. Serum 25(OH)D concentration from Revez et al. GWAS as exposure

We identified a total of 115 independent SNPs for instrumental variables, which collectively accounted for 3.52% of the phenotypic variation (R^2^) in 25(OH)D concentration, as detailed in S1 Table in S2 Appendix. Each SNP chosen had F-statistics above 10, with a median of 43.13 and a range of 35.03–70.86. Following the harmonization process, two SNPs were removed from all analyses due to being palindromic with intermediate allele frequencies, while one additional SNP was removed from the high TSH analysis and two from TPOAb analyses as noted in S2 Table in S2 Appendix. Additionally, SNPs suspected of potential pleiotropy were excluded based on the MR-PRESSO method, as indicated in S5 Table in S2 Appendix. Consequently, the remaining instrumental variants for the MR analysis of serum 25(OH)D concentration with TSH, fT4, fT3, TT3, low TSH, high TSH, autoimmune hypothyroidism, autoimmune hyperthyroidism, TPOAb levels and TPOAb positivity were 113, 110, 106, 111, 110, 113, 112, 105, 107, 38 and 38 respectively, as listed in S7 Table in S2 Appendix.

**Fig 1:**
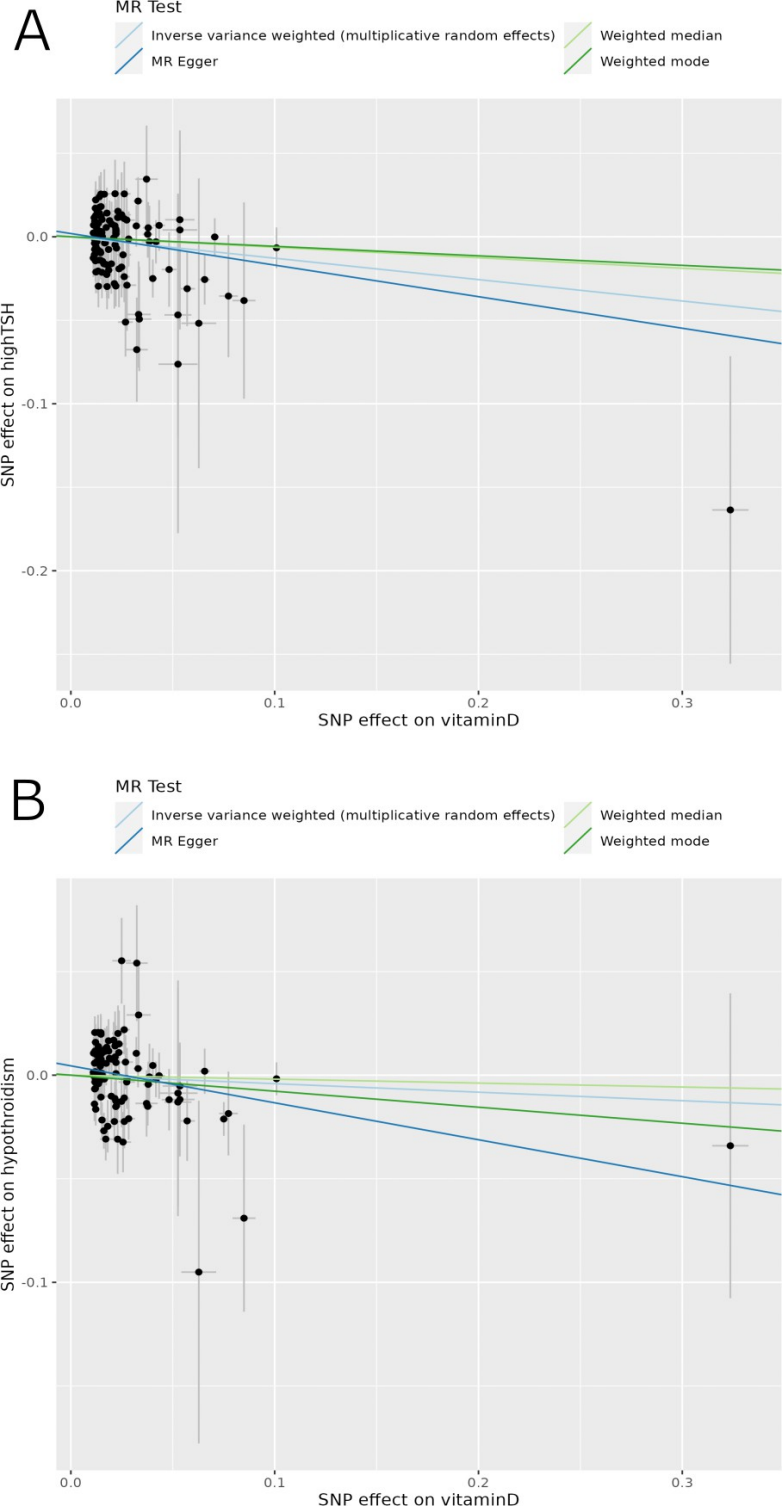
Scatter plots depicting the relationship of A) SNP effects on serum 25(OH)D levels against the SNP effects on high TSH; B) SNP effects on serum 25(OH)D levels against the SNP effects on autoimmune hypothyroidism. MR results were derived from the multiplicative random effects inverse variance weighted (light blue), weighted median (light green), weighted mode (dark green) and MR-Egger (dark blue) method. Each line’s slope represents the estimated causal effect. TSH, thyroid-stimulating hormone.

The multiplicative random-effects IVW MR analysis, as well as MR Egger analysis, showed that genetically predicted serum 25(OH)D concentration was causally associated with high TSH (Table 2 and Fig 1). Each 1 SD increase in serum 25(OH)D concentration was associated with a 12% decrease in the risk of high TSH (p = 0.0197) (Table 2 and S7 Table in S2 Appendix). The Steiger directionality test validated the causal direction of our analysis (S8 Table in S2 Appendix). Additionally, there was no indication of heterogeneity among individual variant estimates (Cochran’s Q = 118.60, p = 0.29) nor evidence of uncorrelated horizontal pleiotropy (MR-PRESSO Global test p = 0.307) (S5 Table in S2 Appendix). The influence of genetically predicted 25(OH)D concentration on high TSH levels showed consistency in direction through both primary and sensitivity MR analyses, despite lacking statistical significance in the weighted median and mode analyses (S7 Table in S2 Appendix). Finally, the direction of the causal link was further validated by CAUSE analysis, which utilized all genome-wide SNPs to demonstrate that the causal model more accurately represented the data compared to the sharing model (delta_elpd = -0.86 < 0). However, the disparity in fit did not achieve statistical significance (p = 0.31) (Table 2 and S1 Appendix–Fig 1).

**Table 2:** Significant results of the MR analysis.

Exposure	Outcome	MR method	N SNPs	OR	p-value	CAUSE delta elpd (p-value)
Vitamin D (Revez)	High TSH	IVW MRE	112	0,880	**0,0197**	-0.86 (0.31)
Vitamin D (Revez)	High TSH	Weighted median	112	0,939	0,504	
Vitamin D (Revez)	High TSH	Weighted mode	112	0,945	0,526	
Vitamin D (Revez)	High TSH	MR Egger	112	0,828	**0,043**	
Vitamin D (Revez)	Autoimmune hypothyroidism	IVW MRE	105	0,960	0,389	-0.56 (0.36)
Vitamin D (Revez)	Autoimmune hypothyroidism	Weighted median	105	0,981	0,768	
Vitamin D (Revez)	Autoimmune hypothyroidism	Weighted mode	105	0,926	0,163	
Vitamin D (Revez)	Autoimmune hypothyroidism	MR Egger	105	0,837	**0,024**	

IVW MRE, inverse variance weighted multiplicative random effects method; MR, mendelian randomization; N, number; OR, odds ratio; SNP, single-nucleotide polymorphism; TSH, thyroid-stimulating hormone.

Additionally, in the MR Egger analysis, genetically predicted 25(OH)D concentration was found to have a suggestive causal link with autoimmune hypothyroidism. Specifically, each 1 SD increase in serum 25(OH)D concentration was associated with a 16.34% decrease in the risk of autoimmune hypothyroidism (p = 0.02) (Table 2 and S7 Table in S2 Appendix).

The Steiger directionality test affirmed the causal direction of our analysis (S8 Table in S2 Appendix).

However, there were indications of heterogeneity between variant estimates (Cochran’s Q = 169.88, p = 3×10^−5^) as well as uncorrelated horizontal pleiotropy (MR-PRESSO Global test p<0.0003), but MR-PRESSO was not able to identify any outliers (S5 Table in S2 Appendix). The effect of genetically predicted 25(OH)D concentration on autoimmune hypothyroidism showed uniformity in direction throughout both primary and sensitivity MR studies, yet failed to reach statistical significance in the multiplicative random-effects IVW, weighted mode, and weighted median analyses (S7 Table in S2 Appendix).

Finally, the direction of the causal link was additionally substantiated by CAUSE analysis. Utilizing all genome-wide SNPs, it indicated that the causal model aligned more closely with the data than the sharing model (delta_elpd = -0.56<0). Nonetheless, the difference in fit did not achieve statistical significance (p = 0.36) (Table 2 and–Fig 2 in S1 Appendix).

## 4. Discussion

Our results support a suggestive causal effect of genetically predicted serum 25(OH)D concentration on high TSH levels and autoimmune hypothyroidism. The results were noted in both frequentist and Bayesian analyses. Each 1 SD increase in genetically predicted serum 25(OH)D concentration was associated with as much as a 12% decrease in the risk of high TSH and a 16.34% decrease in the risk of autoimmune hypothyroidism. We found no causal effect of genetically predicted serum 25(OH)D concentration on reference-range TSH, fT4, fT3, TT3, TPOAb levels or positivity, nor autoimmune hyperthyroidism.

Consistent with existing literature, our findings contribute to the expanding research indicating a significant link between serum 25(OH)D concentration and thyroid function. Studies by Chailurkit et al. [37] and Kim et al. [38] found that higher 25(OH)D concentration was associated with lower TSH levels, particularly in younger individuals, and that low vitamin D status was associated with Hashimoto’s thyroiditis. Similarly, Metwalley et al. [39] and Ma et al. [40] reported that vitamin D deficiency was prevalent in children and adolescents with autoimmune thyroiditis and that a lower 25(OH)D concentration was associated with an increased risk of developing autoimmune thyroid diseases. Additionally, our findings align with those of a large case-control study by Mirhosseini et al. [15], which reported that vitamin D supplementation led to fewer detected cases of hypothyroidism (including both clinical and subclinical forms) and an overall decrease in TSH levels. These findings suggest that maintaining adequate 25(OH)D concentration may play a role in reducing the risk of high TSH levels and autoimmune hypothyroidism. This growing body of observational evidence, paired with our causal results, could provide informed decisions in a clinical setting where vitamin D supplementation can be used as a cost-effective and safe strategy to improve thyroid function and prevent the development of thyroid diseases. Future clinical trials should investigate the therapeutic potential of vitamin D in thyroid disorders while properly accounting for the influence of season, sex and genetic background.

Although mechanisms by which 25(OH)D concentration might affect TSH levels are not fully understood, the study in rat pituitary cells showed that administration of calcitriol affects thyrotropin-releasing hormone (TRH)-induced TSH release [41]. Additionally, studies in rat thyroid cells [42] and FRTL-5 cells [43] showed that calcitriol suppresses TSH-stimulated adenylyl cyclase activity and iodide uptake, respectively. In vivo studies showed that vitamin D affects Dio2 expression. This enzyme, which is necessary for the conversion of T4 into T3, showed an increase in the expression upon vitamin D3 administration in diabetic rats. This was followed by an increase in fT3 levels and a decrease in fT4 levels [44]. However, vitamin D receptor (VDR) knockout mice had only moderately reduced TSH levels, with thyroid physiology not being significantly affected [45].

To date, only two studies using MR methodology investigated the association between serum 25(OH)D concentration and thyroid function. The study conducted by Chen et al. with 10,636 participants from China observed a negative causal relationship between serum 25(OH)D concentration and levels of TPOAb [20]. This causal relationship was not bi-directional since genetically predicted TPOAb levels were not causally affecting serum 25(OH)D concentration [20]. Another study, by Ye et al. in 326,409 Europeans from the UK Biobank, analyzed the causal relationship between serum 25(OH)D concentration and 106 diseases/traits. Among the disorders examined, they found no link between the risk of autoimmune thyroid diseases (hyperthyroidism and hypothyroidism) or thyroid cancer and the genetically predicted serum 25(OH)D concentration using MR technology [19]. Another recent study, published as a preprint [21] has investigated the causal association between serum 25(OH)D concentration and reference-range TSH, fT4 and the risk of hypothyroidism and Hashimoto’s thyroiditis. However, the authors used an earlier GWAS on TSH and fT4 [46] which had 54,288 participants in the TSH GWAS and 49,269 in the fT4 GWAS. Here we utilize the latest published GWAS on these parameters, with 271,040 participants in TSH and 119,120 participants in the fT4 GWAS, which increases the power of the MR study. In addition, the authors [21] used GWAS for hypothyroidism and Hashimoto’s thyroiditis performed in the UK Biobank and FinnGen. This can lead to biased causal estimates because of sample overlap. Ideally, the exposure and outcome datasets in a two-sample MR setting should not be overlapping. Here we utilize the FinnGen GWAS for autoimmune hypothyroidism and hyperthyroidism to avoid sample overlap between the exposure and outcomes.

Certain vitamin D receptor (VDR) gene polymorphisms, namely TaqI (rs731236) and BsmI (rs1544410) have been found to be associated with the risk of autoimmune thyroid diseases [47]. Inclusion of these polymorphisms in the MR analysis would directly violate the Exclusion assumption and would bias the results of MR, as we would have horizontal pleiotropy. In our analysis, these polymorphisms were not present in the set of instruments, therefore they do not represent a potential source of bias.

To the best of our knowledge, our study is the first MR study investigating the association between serum 25(OH)D concentration and fT3, TT3, low TSH and high TSH.

One limitation of our study is that, because of the scarcity of large GWA studies in non-European ancestries, our estimation of causal effects was limited to using only GWAS performed in individuals of European ancestry. This constraint limited our ability to estimate causal effects across diverse ethnic backgrounds. Future research should aim to examine these causal relationships in various ethnic groups. Furthermore, our analysis yielded suggestive causal results only within the analyses utilizing the Revez et al. genome-wide significant SNPs. However, our aim was to provide a comprehensive MR analysis taking into account all the available resources, therefore, we believe that the non-significant findings within the Manousaki et al. analyses will provide valuable insights to researchers investigating this topic.

Furthermore, an investigation of the sex-specific causative effects of vitamin D on thyroid function was outside the scope of our study due to the absence of sex-stratified summary statistics.

In summary, our thorough MR analysis, employing both frequentist and Bayesian methods, provided indications of a potentially beneficial causal effect of serum vitamin D on thyroid disorders. Genetically predicted serum vitamin D levels were found to have a suggestive causal effect on reducing the risk of high TSH and autoimmune hypothyroidism.

## Supporting information

S1 Appendix (DOCX)

S2 Appendix (XLSX)

S3 Appendix STROBE-MR checklist. (DOCX)

## Abbreviations

25(OH)D: 25-hydroxyvitamin D
BMI: body mass index
CAUSE: Causal Analysis Using Summary Effect estimates
fT3: free triiodothyronine
fT4: free thyroxine
GWAS: genome-wide association study
IVW: inverse-variance weighted
MR: Mendelian randomization
MR-PRESSO: Mendelian Randomization Pleiotropy RESidual Sum and Outlier
SNP: single nucleotide polymorphism
TPOAb: thyroid peroxidase antibody
TSH: thyroid-stimulating hormone
TT3: total triiodothyronine

## References

[1] Grundmann M, von Versen-Höynck F. Vitamin D—roles in women’s reproductive health? Reproductive Biology and Endocrinology. 2011;9(1):146. doi: 10.1186/1477-7827-9-146 22047005 PMC3239848

[2] Bikle Daniel D. Vitamin D Metabolism, Mechanism of Action, and Clinical Applications. Chemistry & Biology. 2014;21(3):319–29. doi: 10.1016/j.chembiol.2013.12.016 24529992 PMC3968073

[3] Chakraborti CK. Vitamin D as a promising anticancer agent. Indian J Pharmacol. 2011;43(2):113–20. Epub 2011/05/17. doi: 10.4103/0253-7613.77335 ; PubMed Central PMCID: PMC3081446. 21572642 PMC3081446

[4] Yoav A, Howard A, Yehuda S. Vitamin D and autoimmunity: new aetiological and therapeutic considerations. ANNALS OF THE RHEUMATIC DISEASES. 2007;66(9):1137. doi: 10.1136/ard.2007.069831 17557889 PMC1955167

[5] van Driel M, van Leeuwen JPTM. Vitamin D endocrinology of bone mineralization. Molecular and Cellular Endocrinology. 2017;453:46–51. doi: 10.1016/j.mce.2017.06.008 28606868

[6] Hossein-nezhad A, Spira A, Holick MF. Influence of Vitamin D Status and Vitamin D3 Supplementation on Genome Wide Expression of White Blood Cells: A Randomized Double-Blind Clinical Trial. PloS one. 2013;8(3):e58725. doi: 10.1371/journal.pone.0058725 23527013 PMC3604145

[7] Carlberg C. Vitamin D: A Micronutrient Regulating Genes. CURRENT PHARMACEUTICAL DESIGN. 2019;25(15):1740–6. doi: 10.2174/1381612825666190705193227 31298160

[8] Zimmermann MB, Boelaert K. Iodine deficiency and thyroid disorders. The Lancet Diabetes & Endocrinology. 2015;3(4):286–95. doi: 10.1016/S2213-8587(14)70225-6 25591468

[9] Ventura M, Melo M, Carrilho F. Selenium and Thyroid Disease: From Pathophysiology to Treatment. Int J Endocrinol. 2017;2017:1297658. doi: 10.1155/2017/1297658 28255299 PMC5307254

[10] Liu F, Wang K, Nie J, Feng Q, Li X, Yang Y, et al. Relationship between dietary selenium intake and serum thyroid function measures in U.S. adults: Data from NHANES 2007–2012. FRONTIERS IN NUTRITION. 2022;9. doi: 10.3389/fnut.2022.1002489 36299994 PMC9589160

[11] Winther KH, Rayman MP, Bonnema SJ, Hegedüs L. Selenium in thyroid disorders—essential knowledge for clinicians. Nature Reviews Endocrinology. 2020;16(3):165–76. doi: 10.1038/s41574-019-0311-6 32001830

[12] Zhao J, Wang H, Zhang Z, Zhou X, Yao J, Zhang R, et al. Vitamin D deficiency as a risk factor for thyroid cancer: A meta-analysis of case-control studies. Nutrition. 2019;57:5–11. doi: 10.1016/j.nut.2018.04.015 30086436

[13] Taheriniya S, Arab A, Hadi A, Fadel A, Askari G. Vitamin D and thyroid disorders: a systematic review and Meta-analysis of observational studies. BMC Endocrine Disorders. 2021;21(1):171. doi: 10.1186/s12902-021-00831-5 34425794 PMC8381493

[14] Babić Leko M, Jureško I, Rozić I, Pleić N, Gunjača I, Zemunik T. Vitamin D and the Thyroid: A Critical Review of the Current Evidence. International Journal of Molecular Sciences [Internet]. 2023; 24(4). doi: 10.3390/ijms24043586 36835005 PMC9964959

[15] Mirhosseini N, Brunel L, Muscogiuri G, Kimball S. Physiological serum 25-hydroxyvitamin D concentrations are associated with improved thyroid function—observations from a community-based program. ENDOCRINE. 2017;58(3):563–73. doi: 10.1007/s12020-017-1450-y 29067607 PMC5693977

[16] Waterhouse M, Pham H, Rahman ST, Baxter C, Duarte Romero B, Armstrong BK, et al. The Effect of Vitamin D Supplementation on Hypothyroidism in the Randomized Controlled D-Health Trial. Thyroid®. 2023;33(11):1302–10. doi: 10.1089/thy.2023.0317 37698908

[17] Davey Smith G, Ebrahim S. ‘Mendelian randomization’: can genetic epidemiology contribute to understanding environmental determinants of disease?*. International Journal of Epidemiology. 2003;32(1):1–22. doi: 10.1093/ije/dyg070 12689998

[18] Pingault J-B, O’Reilly PF, Schoeler T, Ploubidis GB, Rijsdijk F, Dudbridge F. Using genetic data to strengthen causal inference in observational research. Nature Reviews Genetics. 2018;19(9):566–80. doi: 10.1038/s41576-018-0020-3 29872216

[19] Ye Y, Yang H, Wang Y, Zhao H. A comprehensive genetic and epidemiological association analysis of vitamin D with common diseases/traits in the UK Biobank. Genetic Epidemiology. 2021;45(1):24–35. doi: 10.1002/gepi.22357 32918767

[20] Chen Y, Han B, Zhu C, Li Q, Chen C, Zhai H, et al. Bidirectional Mendelian Randomization Analysis for Vitamin D and Thyroid Peroxidase Antibody. Int J Endocrinol. 2022;2022:2260388. doi: 10.1155/2022/2260388 35399300 PMC8993571

[21] Mahdi A, Sahand Tehrani F, Aysan M, Danial H, Amir Hossein G, Amir Hesam S, et al. Genetically predicted 25-Hydroxyvitamin D levels on Hypothyroidism: A two-sample Mendelian Randomization. medRxiv: the preprint server for health sciences. 2023:2023.08.30.23294811. doi: 10.1101/2023.08.30.23294811

[22] Pleić N, Gunjača I, Babić Leko M, Zemunik T. Thyroid Function and Metabolic Syndrome: A Two-Sample Bidirectional Mendelian Randomization Study. The Journal of Clinical Endocrinology & Metabolism. 2023;108(12):3190–200. doi: 10.1210/clinem/dgad371 37339283

[23] Manousaki D, Mitchell R, Dudding T, Haworth S, Harroud A, Forgetta V, et al. Genome-wide Association Study for Vitamin D Levels Reveals 69 Independent Loci. The American Journal of Human Genetics. 2020;106(3):327–37. doi: 10.1016/j.ajhg.2020.01.017 32059762 PMC7058824

[24] Revez JA, Lin T, Qiao Z, Xue A, Holtz Y, Zhu Z, et al. Genome-wide association study identifies 143 loci associated with 25 hydroxyvitamin D concentration. Nat Commun. 2020;11(1):1647. doi: 10.1038/s41467-020-15421-7 32242144 PMC7118120

[25] Sterenborg RBTM, Steinbrenner I, Li Y, Bujnis MN, Naito T, Marouli E, et al. Multi-trait analysis characterizes the genetics of thyroid function and identifies causal associations with clinical implications. Nat Commun. 2024;15(1):888. doi: 10.1038/s41467-024-44701-9 38291025 PMC10828500

[26] Medici M, Porcu E, Pistis G, Teumer A, Brown SJ, Jensen RA, et al. Identification of Novel Genetic Loci Associated with Thyroid Peroxidase Antibodies and Clinical Thyroid Disease. Plos Genet. 2014;10(2):e1004123. doi: 10.1371/journal.pgen.1004123 24586183 PMC3937134

[27] Kurki MI, Karjalainen J, Palta P, Sipilä TP, Kristiansson K, Donner KM, et al. FinnGen provides genetic insights from a well-phenotyped isolated population. Nature. 2023;613(7944):508–18. doi: 10.1038/s41586-022-05473-8 36653562 PMC9849126

[28] Matushyn M, Bose M, Mahmoud AA, Cuthbertson L, Tello C, Bircan KO, et al. SumStatsRehab: an efficient algorithm for GWAS summary statistics assessment and restoration. BMC Bioinformatics. 2022;23(1):443. doi: 10.1186/s12859-022-04920-7 36284273 PMC9594936

[29] Verbanck M, Chen C-Y, Neale B, Do R. Detection of widespread horizontal pleiotropy in causal relationships inferred from Mendelian randomization between complex traits and diseases. Nature genetics. 2018;50(5):693–8. doi: 10.1038/s41588-018-0099-7 29686387 PMC6083837

[30] Burgess S, Butterworth A, Thompson SG. Mendelian Randomization Analysis With Multiple Genetic Variants Using Summarized Data. Genetic Epidemiology. 2013;37(7):658–65. doi: 10.1002/gepi.21758 24114802 PMC4377079

[31] Bowden J, Del Greco M F, Minelli C, Davey Smith G, Sheehan N, Thompson J. A framework for the investigation of pleiotropy in two-sample summary data Mendelian randomization. Statistics in Medicine. 2017;36(11):1783–802. doi: 10.1002/sim.7221 28114746 PMC5434863

[32] Bowden J, Davey Smith G, Haycock PC, Burgess S. Consistent Estimation in Mendelian Randomization with Some Invalid Instruments Using a Weighted Median Estimator. Genetic Epidemiology. 2016;40(4):304–14. doi: 10.1002/gepi.21965 27061298 PMC4849733

[33] Hartwig FP, Davey Smith G, Bowden J. Robust inference in summary data Mendelian randomization via the zero modal pleiotropy assumption. International Journal of Epidemiology. 2017;46(6):1985–98. doi: 10.1093/ije/dyx102 29040600 PMC5837715

[34] R Core Team. R: A Language and Environment for Statistical Computing. 2016.

[35] Hemani G, Tilling K, Davey Smith G. Orienting the causal relationship between imprecisely measured traits using GWAS summary data. Plos Genet. 2017;13(11):e1007081. doi: 10.1371/journal.pgen.1007081 29149188 PMC5711033

[36] Morrison J, Knoblauch N, Marcus JH, Stephens M, He X. Mendelian randomization accounting for correlated and uncorrelated pleiotropic effects using genome-wide summary statistics. Nature genetics. 2020;52(7):740–7. doi: 10.1038/s41588-020-0631-4 32451458 PMC7343608

[37] Chailurkit L-o, Aekplakorn W, Ongphiphadhanakul B. High Vitamin D Status in Younger Individuals Is Associated with Low Circulating Thyrotropin. Thyroid®. 2012;23(1):25–30. doi: 10.1089/thy.2012.0001 22931506

[38] Kim D. Low vitamin D status is associated with hypothyroid Hashimoto’s thyroiditis. Hormones. 2016;15(3):385–93. doi: 10.14310/horm.2002.1681 27394703

[39] Metwalley KA, Farghaly HS, Sherief T, Hussein A. Vitamin D status in children and adolescents with autoimmune thyroiditis. Journal of Endocrinological Investigation. 2016;39(7):793–7. doi: 10.1007/s40618-016-0432-x 26809977

[40] Ma J, Wu D, Li C, Fan C, Chao N, Liu J, et al. Lower Serum 25-Hydroxyvitamin D Level is Associated With 3 Types of Autoimmune Thyroid Diseases. Medicine. 2015;94(39). doi: 10.1097/MD.0000000000001639 26426654 PMC4616844

[41] D′Emden MC, Wark JD. 1,25-DIHYDROXYVITAMIN D3 ENHANCES THYROTROPIN RELEASING HORMONE INDUCED THYROTROPIN SECRETION IN NORMAL PITUITARY CELLS. Endocrinology. 1987;121(3):1192–4. doi: 10.1210/endo-121-3-1192 3113918

[42] Berg JP, Liane KM, Bjørhovde SB, Bjøro T, Torjesen PA, Haug E. Vitamin D receptor binding and biological effects of cholecalciferol analogues in rat thyroid cells. The Journal of Steroid Biochemistry and Molecular Biology. 1994;50(3):145–50. doi: 10.1016/0960-0760(94)90021-3 8049143

[43] Lamberg-Allardt C, Valtonen E, Polojärvi M, Stewen P. Characterization of a 1,25-dihydroxy-vitamin D3 receptor in FRTL-5 cells. Evidence for an inhibitory effect of 1,25-dihydroxy-vitamin D3 on thyrotropin-induced iodide uptake. Molecular and Cellular Endocrinology. 1991;81(1):25–31. doi: 10.1016/0303-7207(91)90201-3 1665829

[44] Alrefaie Z, Awad H. Effect of vitamin D3 on thyroid function and de-iodinase 2 expression in diabetic rats. ARCHIVES OF PHYSIOLOGY AND BIOCHEMISTRY. 2015;121(5):206–9. doi: 10.3109/13813455.2015.1107101 26599099

[45] Clinckspoor I, Gérard A-C, Van Sande J, Many M-C, Verlinden L, Bouillon R, et al. The Vitamin D Receptor in Thyroid Development and Function. European Thyroid Journal. 2012;1(3):168–75. doi: 10.1159/000342363 24783016 PMC3821476

[46] Teumer A, Chaker L, Groeneweg S, Li Y, Di Munno C, Barbieri C, et al. Genome-wide analyses identify a role for SLC17A4 and AADAT in thyroid hormone regulation. Nat Commun. 2018;9(1):4455. doi: 10.1038/s41467-018-06356-1 30367059 PMC6203810

[47] Feng M, Li H, Chen S-F, Li W-F, Zhang F-B. Polymorphisms in the vitamin D receptor gene and risk of autoimmune thyroid diseases: a meta-analysis. ENDOCRINE. 2013;43(2):318–26. doi: 10.1007/s12020-012-9812-y 23065592

