# Supplementary material for: Vitamin D and thyroid function: A mendelian randomization study
Source: PLoS One. 2024 Jun 20;19(6):e0304253. doi: 10.1371/journal.pone.0304253 (PMC11189194; doi:10.1371/journal.pone.0304253)
Supplement: S1 Appendix — (DOCX) [file pone.0304253.s001.docx]

**Supplementary file 1**

TABLE OF CONTENTS

1. Data sources

1.1. Manousaki et al. - summary association data for serum 25-hydroxyvitamin D [25(OH)D] concentration

1.2. Revez et al. - summary association data for serum 25(OH)D concentration

1.3. Sterenborg et al. – The ThyroidOmics consortium summary association data for thyroid function parameters

1.4. Medici et al. - The ThyroidOmics consortium summary association data for thyroid peroxidase antibody (TPOAb) levels and positivity

1.5. The FinnGen consortium – summary association data for autoimmune hypothyroidism and autoimmune hyperthyroidism

2. Supplementary Figures

- Supplementary Figure 1. Results of the CAUSE analysis for the genetically predicted effect of serum 25(OH)D concentration on high TSH.
- Supplementary Figure 2. Results of the CAUSE analysis for the genetically predicted effect of serum 25(OH)D concentration on autoimmune hypothyroidism.

3. Supplementary References

**1. Data sources**

1.1. Manousaki et al. - summary association data for serum 25(OH)D concentration [[1](#_ENREF_1)]

Manousaki et al. utilized the UK Biobank, where concentrations of 25(OH)D (nmol/L) was measured using the Diasorin assay, obtained from 465,415 samples, which corresponded to 449,978 participants in the UK Biobank. These measurements were taken either during the initial assessment phase (2006–2010) or at the first follow-up (2012–2013). Manousaki et al. [[1](#_ENREF_1)] used baseline 25(OH)D measurements from 401,460 individuals from the white British subset of the UK Biobank.

Within this study, to consider the impact of vitamin D supplement usage, adjustments were made to the 25(OH)D levels. Specifically, 21.2 nmol/L was subtracted from the 25(OH)D levels of 24,874 participants who used vitamin D supplements, accounting for 6% of the study cohort. This subtraction was based on the average increase in 25(OH)D levels from consuming 400 IU of cholecalciferol daily, a common amount in vitamin D supplements. In cases where 25(OH)D levels fell below 10 nmol/L (the Diasorin assay's detection limit) after this adjustment, particularly in 3,057 individuals using vitamin D supplements, levels were set to 10 nmol/L.

The 25(OH)D levels were then log-transformed and standardized to a mean of 0 and a standard deviation of 1.

Following rigorous quality control, 20,370,874 genetic variants from the autosomes and the X chromosome in the UK Biobank data, imputed to the combined Haplotype Reference Consortium (HRC) and UK10K panel, were used to assess their association with 25(OH)D levels. Variants retained met criteria of a minor allele frequency (MAF) > 1%, an imputation quality score > 0.3, and a Hardy-Weinberg p-value > 1 × 10^-6^.

The Manousaki et al. genome-wide association study (GWAS) [[1](#_ENREF_1)] involved assessing the additive effects of single nucleotide polymorphisms (SNPs) on 25(OH)D levels using a linear mixed-model in the BOLT-LMM software. The authors fitted the model on hard-called genotypes from 488,377 participants, which encompassed 803,113 SNPs. The model included several covariates: age, sex, the season when the 25(OH)D measurement was taken (categorized as 1 for winter [January to March], 2 for spring [April to June], 3 for summer [July to September], and 4 for fall [October to December]), genotype batch, the genotype array, and the assessment center, which served as a proxy for geographical latitude. The authors set a p-value threshold at 6.6 × 10^-9^ as suitable for genome-wide significance in the analysis of UK Biobank data, taking into account multiple testing.

The authors conducted a comparison between the GWAS results from the UK Biobank and those from a previous 25(OH)D GWAS published by their group, which included 42,274 samples of European ancestry. Following this, they merged the summary-level results of these two GWASs in an inverse variance-weighted fixed-effects meta-analysis using the GWAMA software.

In both GWASs, 25(OH)D levels were adjusted for age, sex, genotyping center, and season of measurement. In the earlier GWAS, adjustments were also made for body mass index (BMI). However, in the UK Biobank GWAS, BMI was not used as an adjustment factor to avoid introducing collider bias, given its heritable nature. Additionally, the UK Biobank GWAS included adjustments for vitamin D supplementation, as this information was available for all participants, unlike in the earlier 25(OH)D GWAS.

1.2. Revez et al. - summary association data for serum 25(OH)D concentration [[2](#_ENREF_2)]

Revez et al. [[2](#_ENREF_2)] also utilized the UK Biobank (UKB) data. These participants, all registered with the National Health Service, were located approximately 25 miles from any of the 22 recruitment centers across the UK and were enrolled between 2006 and 2010.

The UKB group conducted quality control on the genotype data and imputed it to the Haplotype Reference Consortium (HRC) and UK10K reference panels.

The authors extracted variants with a minor allele count (MAC) greater than 5 and an imputation score above 0.3 for all individuals. These genotype probabilities were then converted to hard-call genotypes using PLINK2. Variants were excluded if they had more than 5% genotype missingness, a Hardy–Weinberg equilibrium test p-value greater than 1 × 10^-5^, or a MAF less than 0.01. Consequently, 8,806,780 variants, including SNPs and potentially small insertion/deletions (INDELs), with 260,713 SNPs on the X chromosome, were available for analysis.

As already mentioned for Manousaki et al.[[1](#_ENREF_1)], 25(OH)D levels in the participants were determined from blood samples taken during two key periods: the initial assessment phase between 2006 and 2010, and a subsequent assessment between 2012 and 2013. The measurement of 25(OH)D was carried out using the Diasorin Liason®, a chemiluminescent immunoassay (CLIA), which quantifies the total concentration of 25(OH)D, encompassing both 25(OH)D3 and 25(OH)D2. Participants whose 25(OH)D concentrations fell outside the assay's validated range (10–375 nmol L^-1^) were not included in the study. The average within-laboratory coefficient of variation (CV) (and standard deviation) for this assay varied between 5.04 (4.73) and 6.14 (2.21). Rvez et al., unlike Manousaki et al., did not correct the 25(OH)D levels for the use of vitamin D supplements.

Out of 502,536 participants in the UK Biobank (UKB), 449,978 (approximately 90%) had their 25(OH)D levels measured, with the majority of these measurements (448,376, or 99.6%) coming from the initial assessment visit. The Revez et al.[[2](#_ENREF_2)] analysis focused on 417,580 individuals of European ancestry who had available 25(OH)D concentrations, out of which 318,851 were unrelated (identified using gcta with a relation cut-off of 0.05).

In order to identify genetic variants linked to 25(OH)D levels, a linear mixed model GWAS was conducted using fastGWA. This tool, part of the GCTA software suite, employs a mixed linear model (MLM) approach for GWAS using a sparse genomic relationship matrix (GRM) to account for genetic structure within large datasets like the UK Biobank. This sparse GRM was generated for UK Biobank participants of European ancestry using HapMap3 SNPs.

This study [[2](#_ENREF_2)] used a rank-based inverse-normal transformation (RINT) for the 25(OH)D levels and included several covariates in the model: age at the time of assessment, sex, month of assessment, assessment center, information on supplement intake, genotyping batch, and the first 40 ancestry principal components (PCs).

Furhermore, the largest GWAS for 25(OH)D conducted up until the publication of Revez et al. GWAS was by the SUNLIGHT consortium and used BMI as a covariate. Consequently, the UK Biobank (UKB) results by Revez et al. were also generated with BMI included in the model for use in meta-analysis. Furthermore, the UKB GWAS results utilized for meta-analysis were distinct from the reported GWAS findings in that they applied a natural-log transformation to 25(OH)D levels and did not incorporate supplement intake as a covariate. Prior to the meta-analysis, the SUNLIGHT summary statistics, covering 2,579,297 SNPs, were imputed using ImpG. Subsequent to data management processes, a sample size-based method was employed to carry out the meta-analysis on 6,912,294 SNPs that were common between the datasets. Evethough the authors in Revez et al. pefrormed the meta-analysis with the SUNLIGHT data, given that the meta-analysis only increased the total number of significant loci by seven, and given their preference not to include BMI as a covariate, the authors continued with the UKB-only results for their downstream analyses.

1.3. Sterenborg et al. - The ThyroidOmics consortium - summary association data for thyroid function parameters [[3](#_ENREF_3)]

The ThyroidOmics Consortium invited cohorts composed of individuals of European descent to participate in the study. The study excluded individuals under 18 years old, those of non-European descent, anyone using thyroid medication (specifically those categorized under the Anatomical Therapeutic Chemical (ATC) code H03), and those with a history of thyroid surgery. Data was collected on the gender distribution, average age, and thyroid hormone levels from all participating cohorts. Only studies with a minimum of 40 cases were included in the analyses for high and low thyroid-stimulating hormone (TSH) levels, with individuals within the TSH reference range serving as controls.

Reference ranges for TSH were typically determined by considering the upper and lower 2.5% percentiles of the TSH distribution, factoring in characteristics of both the assay and the population, as well as other environmental influences like iodine levels in the population. In cases where this data wasn't available, the TSH reference range provided by the assay manufacturer was used. TSH, free thyroxine (fT4), free triiodothyronine (fT3), and total triiodothyronine (TT3) were treated as continuous variables and underwent an inverse normal transformation for analysis. Individuals with TSH levels exceeding the upper or falling below the lower limit of the reference range set by their cohort were identified as cases for the high and low TSH GWAS, respectively. Those with TSH levels within the cohort-specific reference range were classified as controls.

In all the studies, genotyping was conducted using genome-wide arrays. The genomic data were then imputed to either the Haplotype Reference Consortium (HRC) version 1.1, or the 1000 Genomes Project phase 1 or 3. Each study employed a multiple linear regression model with an additive genetic effect to analyze the association between phenotype and genotype for continuous traits, and logistic regression for binary traits. These models were adjusted for variables such as sex, age, and the square of age (to consider non-linear age effects), along with other relevant cohort-specific factors. These additional factors included principal components to adjust for population stratification, family structure in family-based studies, study site, village, field center, or laboratory batch as appropriate.

Before conducting the meta-analyses, genetic variants that had MAF of 5% or less, or an imputation quality score of 0.4 or lower, were removed. The meta-analyses were carried out by two separate analysts using the inverse variance-weighted method and a fixed-effect model approach, utilizing the METAL software package.

1.4. Medici et al. - The ThyroidOmics consortium summary association data for thyroid peroxidase antibody (TPOAb) levels and positivity [[4](#_ENREF_4)]

In the Medici et al. GWAS[[4](#_ENREF_4)] for thyroid peroxidase antibody (TPOAb) levels and positivity, participants were enlisted from 16 separate studies, which included both community-based and family studies.

Serum levels of TPOAb were measured using various assays. Subjects were classified as TPOAb-positive if their TPOAb levels exceeded the cut-off for TPOAb-positivity specified for each assay by its manufacturer.

Samples underwent genotyping using various genotyping arrays. Each study conducted its sample and SNP quality control procedures. For every GWAS, over 2.5 million SNPs were imputed based on the CEU samples from Phase 2 of the International HapMap Project.

Two GWAS were conducted: one on TPOAb positivity and another on continuous TPOAb levels. Participants using thyroid medication were excluded. Logistic regression analyses were utilized to test each SNP for its association with TPOAb-positivity, adjusting for age and sex. In cohorts with family structures, a linear mixed model, adjusted for age and sex, approximated the probability of being affected. This model predicted the expected proportion of "risk" (effective) alleles in cases and controls, aiding in odds ratio estimation. For the SardiNIA cohort, analysis was limited to unrelated individuals. In the GWAS for continuous TPOAb levels, samples below the minimum detection limit of the TPOAb assay were not considered. TPOAb levels were log-transformed, and sex-specific, age-adjusted standardized residuals were calculated. The association of each SNP with these residuals was then tested using linear regression analyses (additive model), with adjustments for relatedness in family-structured studies.

Prior to the meta-analysis, SNPs with a MAF less than 1% or poor imputation quality were excluded. The results from each GWAS were then combined using a population size weighted z-score based meta-analysis, utilizing the METAL software.

1.5. The FinnGen consortium – summary association data for autoimmune hypothyroidism and autoimmune hyperthyroidism[[5](#_ENREF_5)]

Autoimmune hypothyroidism and autoimmune hyperthyroidism GWAS summary statistics were obtained from the FinnGen consortium [[5](#_ENREF_5)]. The FinnGen study is a large-scale genomics initiative that has analyzed over 500,000 Finnish biobank samples and correlated genetic variation with health data to understand disease mechanisms and predispositions. The phenotypes used in this study were “hypothyroidism, strict autoimmune” and “autoimmune hyperthyroidism”. FinnGen GWAS on hypothyroidism included 287,247 Finnish adults, of which 36,321 were cases and 250,926 were controls. FinnGen GWAS on hyperthyroidism included 257,552 Finnish adults, of which 1,621 were cases and 255,931 were controls. Participants classified as having autoimmune hypothyroidism included those who already received treatment, however, the same information was not present for participants in the autoimmune hyperthyroidism group.

**2. Supplementary Figures**


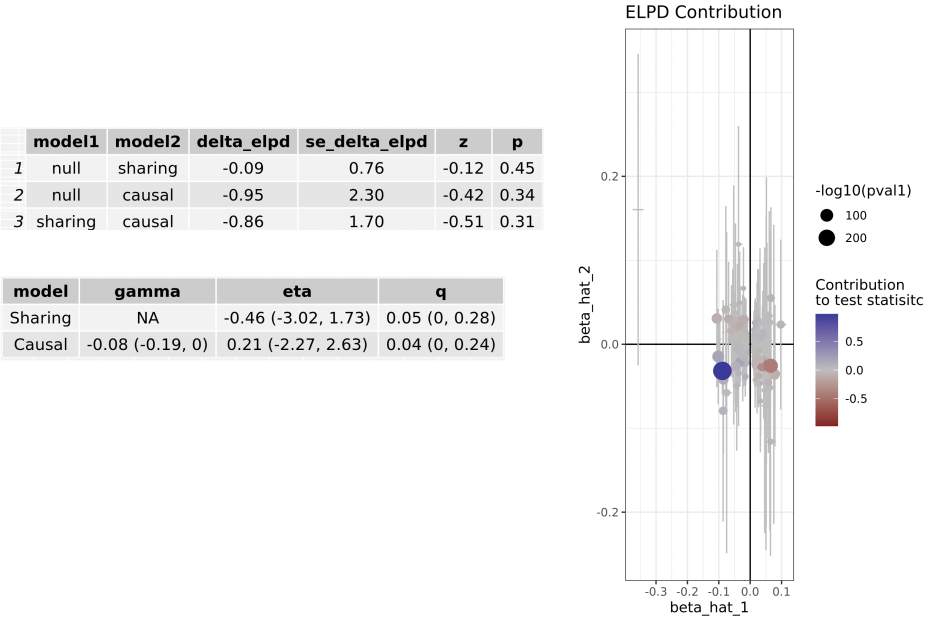


Supplementary Figure 1. Results of the CAUSE analysis for the genetically predicted effect of serum 25(OH)D concentration on high TSH.

Models are compared using the delta_elpd statistic. A negative delta_elpd indicates that model2 is a better fit to data than model1. A negative delta_elpd in the 3^rd^ row of the upper table suggests that the causal model is a better fit to data than the sharing model. Column z represents a z-score that can be compared to a normal distribution to test if the difference in model fit is significant. The p column represents the corresponding p-value. The bottom table represents the estimated sharing and causal effects. Eta represents the effect of the sharing factor (a shared factor affects both serum 25(OH)D concentration and high TSH). Gamma represents the causal factor effect, “-0.08 (-0.19, 0)” which is the genetically predicted effect of serum 25(OH)D concentration on high TSH after adjusting for both correlated and uncorrelated horizontal pleiotropy. The ELPD contribution plot visually represents the contribution of each SNP to the test statistic. The plot shows only genome-wide significant SNPs. Warmer tones indicate a contribution to the causal model, while colder tones indicate a contribution to the sharing model.

**
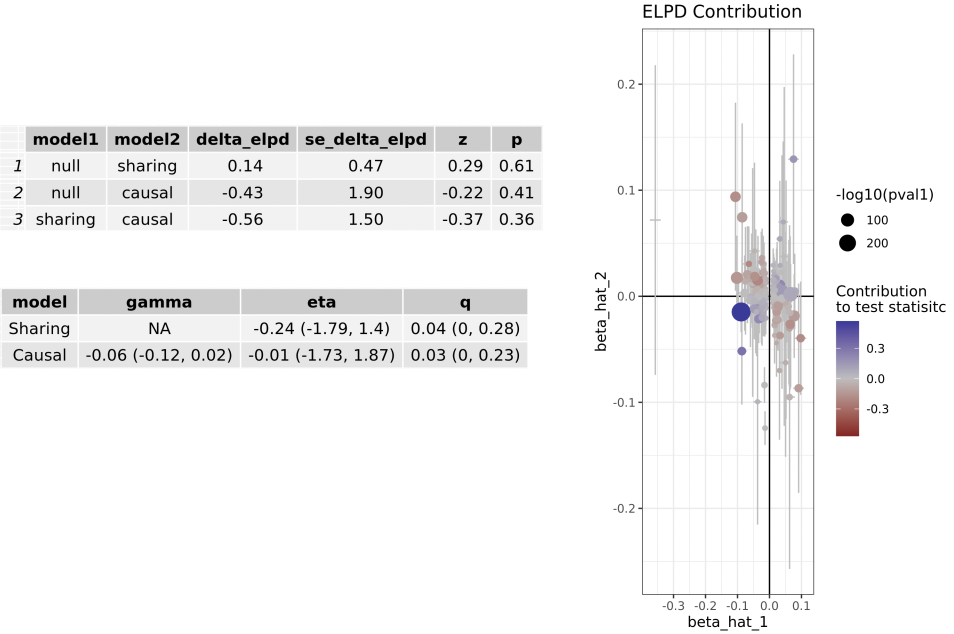
**

Supplementary Figure 2. Results of the CAUSE analysis for the genetically predicted effect of serum 25(OH)D concentration on autoimmune hypothyroidism.

Models are compared using the delta_elpd statistic. A negative delta_elpd indicates that model2 is a better fit to data than model1. A negative delta_elpd in the 3^rd^ row of the upper table suggests that the causal model is a better fit to data than the sharing model. Column z represents a z-score that can be compared to a normal distribution to test if the difference in model fit is significant. The p column represents the corresponding p-value. The bottom table represents the estimated sharing and causal effects. Eta represents the effect of the sharing factor (a shared factor affects both serum 25(OH)D concentration and autoimmune hypothyroidism). Gamma represents the causal factor effect, “-0.06 (-0.12, 0.02)” which is the genetically predicted effect of serum 25(OH)D concentration on autoimmune hypothyroidism after adjusting for both correlated and uncorrelated horizontal pleiotropy. The ELPD contribution plot visually represents the contribution of each SNP to the test statistic. The plot shows only genome-wide significant SNPs. Warmer tones indicate a contribution to the causal model, while colder tones indicate a contribution to the sharing model.

**3. Supplementary references**

1. Manousaki D, Mitchell R, Dudding T, Haworth S, Harroud A, Forgetta V, et al. Genome-wide Association Study for Vitamin D Levels Reveals 69 Independent Loci. The American Journal of Human Genetics. 2020;106(3):327-37. doi: https://doi.org/10.1016/j.ajhg.2020.01.017.

2. Revez JA, Lin T, Qiao Z, Xue A, Holtz Y, Zhu Z, et al. Genome-wide association study identifies 143 loci associated with 25 hydroxyvitamin D concentration. Nat Commun. 2020;11(1):1647. doi: 10.1038/s41467-020-15421-7.

3. Sterenborg RBTM, Steinbrenner I, Li Y, Bujnis MN, Naito T, Marouli E, et al. Multi-trait analysis characterizes the genetics of thyroid function and identifies causal associations with clinical implications. Nat Commun. 2024;15(1):888. doi: 10.1038/s41467-024-44701-9.

4. Medici M, Porcu E, Pistis G, Teumer A, Brown SJ, Jensen RA, et al. Identification of Novel Genetic Loci Associated with Thyroid Peroxidase Antibodies and Clinical Thyroid Disease. Plos Genet. 2014;10(2):e1004123. doi: 10.1371/journal.pgen.1004123.

5. Kurki MI, Karjalainen J, Palta P, Sipilä TP, Kristiansson K, Donner KM, et al. FinnGen provides genetic insights from a well-phenotyped isolated population. Nature. 2023;613(7944):508-18. doi: 10.1038/s41586-022-05473-8.
